# Terpinen-4-ol Improves Lipopolysaccharide-Induced Macrophage Inflammation by Regulating Glutamine Metabolism

**DOI:** 10.3390/foods13121842

**Published:** 2024-06-12

**Authors:** Yanhui Liu, Xin Tang, Huazhen Zhang, Linyan Zheng, Ping Lai, Chang Guo, Jingfan Ma, Hongbo Chen, Longxin Qiu

**Affiliations:** 1 The Department of Biological Sciences and Biotechnology, College of Life Sciences, Longyan University, Longyan 364012, China; yanhuiliu520@gmail.com (Y.L.); zhz17759786160@163.com (H.Z.); 18760168869@163.com (L.Z.); 18250113158@163.com (P.L.); 82015017@lyun.edu.cn (C.G.); m_jingfan@163.com (J.M.); 2 Key Laboratory of Preventive Veterinary Medicine and Biotechnology, Longyan University, Longyan 364012, China; 3 The Department of Veterinary Medicine and Animal Science, College of Life Sciences, Longyan University, Longyan 364012, China; tangxin22417@163.com; 4 Fujian Provincial Key Laboratory for the Prevention and Control of Animal Infectious Diseases and Biotechnology, Longyan 364012, China

**Keywords:** terpinen-4-ol, lipopolysaccharide (LPS), inflammatory cytokines, non-targeted metabolomics, glutamine

## Abstract

Terpinen-4-ol (T-4-O) is an important component of tea tree oil and has anti-inflammatory effects. Currently, there are very few studies on the mechanisms by which T-4-O improves lipopolysaccharide (LPS)-induced macrophage inflammation. In this study, LPS-stimulated mouse RAW264.7 macrophages were used as a model to analyze the effects of T-4-O on macrophage inflammatory factors and related metabolic pathways in an inflammatory environment. The results showed that T-4-O significantly decreased the expression levels of inflammatory cytokines induced by LPS. Cellular metabolism results showed that T-4-O significantly decreased the ratio of the extracellular acidification rate and oxygen consumption rate. Non-targeted metabolomics results showed that T-4-O mainly affected glutamine and glutamate metabolism and glycine, serine, and threonine metabolic pathways. qPCR results showed that T-4-O increased the transcript levels of GLS and GDH and promoted glutamine catabolism. Western blotting results showed that T-4-O inhibited the mTOR and IκB, thereby decreasing NF-κB activity. The overall results showed that T-4-O inhibited mTOR phosphorylation to promote glutamine metabolism and increased cell oxidative phosphorylation levels, thereby inhibiting the expression of LPS-induced inflammatory cytokines.

## 1. Introduction

Macrophages participate in all stages of immune responses and are the first line of defense in the immune system. When a pathogen invades the body, macrophages secrete inflammatory mediators, such as nitric oxide (NO), tumor necrosis factor α (*TNF-α*), and interleukin-6 (*IL-6*), to induce cellular immunity and kill pathogenic microorganisms. Inflammatory responses can promote the body to overcome noxious stimuli, repair tissue, and restore homeostasis [1]. However, inflammatory responses also have adverse outcomes, such as immune imbalances, further tissue injury, sepsis, and organ failure. Therefore, it is important to search for anti-inflammatory drugs that reduce adverse reactions caused by inflammation [2]. Lipopolysaccharide (LPS) is an important component of the cell wall outer membrane of Gram-negative bacteria and is considered to be the main cause of systemic inflammatory response syndrome [3]. LPS is an important pattern recognition ligand that binds to Toll-like receptor 4 (TLR-4) proteins on macrophage surfaces [4] and exerts its effects through the nuclear factor κB (NF-κB) signaling pathway, thereby inducing the expression of pro-inflammatory cytokines [5]. Therefore, LPS-induced macrophages are often used to establish a cellular model of inflammation and immunomodulation.

Tea tree oil (TTO) is a colorless to pale yellow oily liquid that is obtained by steam distillation of the fresh leaves of *Melaleuca alternifolia* and has a unique camphor aroma and mint-like freshness [6]. This essential oil has broad antibacterial, anti-inflammatory, antiviral, antioxidant, and anti-insect characteristics and is widely used in food additive fields such as fruit and vegetable preservation, meat processing, and beverage production [7]. TTO is a complex mixture with more than 100 components, such as monoterpenes, sesquiterpenes, and phenols [8]. Most of these components are terpenes, which account for 80–90% of TTO. The main terpenes include terpinen-4-ol (T-4-O), α-terpineol, and 1,8-cineole. The T-4-O content of TTO is at least 30%, and this substance is considered to be the main anti-inflammatory and antibacterial active ingredient in TTO [7,9,10]. Studies have reported that T-4-O shows antioxidant and anti-inflammatory effects in LPS-stimulated human monocytes [11,12]. However, there are very few studies on the mechanisms whereby T-4-O improves LPS-induced macrophage inflammation. In this study, changes in metabolites, enriched pathways, and possible regulatory mechanisms of T-4-O on LPS-induced macrophage inflammation were studied to provide a theoretical basis for anti-inflammatory research and application of the monoterpene T-4-O from TTO.

## 2. Materials and Methods

### 2.1. Cell Lines

RAW264.7 cells were obtained from the National Collection of Authenticated Cell Cultures, Chinese Academy of Sciences and cultured in Dulbecco’s Modified Eagle Medium (DMEM) supplemented with 10% fetal bovine serum (FBS), 100 IU/mL penicillin, and 100 mg/mL streptomycin at 37 °C with 5% CO_2_.

### 2.2. Reagents and Antibodies

A CCK-8 assay kit, TRIzol reagent, reverse transcription kit, and SYBR green PCR master mix were ordered from Vazyme (Nanjing, China). LPS and T-4-O were purchased from Sigma-Aldrich (St. Louis, MO, USA). Polypropylene fluoride membranes were ordered from Millipore (Billerica, MA, USA). Bicinchoninic acid protein assay kits, RIPA lysis buffer, and a phenylmethylsulfonyl fluoride protease inhibitor were ordered from Beyotime (Shanghai, China). The antibodies anti-mTOR, anti-p-mTOR, anti-AMPK, anti-p-AMPK, anti-NF-κB-p65, anti-p-NF-κB-p65, anti-IκBα, and anti-p-IκBα were ordered from Cell Signaling Technology (Danvers, MA, USA). The antibodies anti-α-tubulin and anti-GAPDH were ordered from Proteintech (Wuhan, China). Horseradish peroxidase (HRP)-conjugated goat anti-mouse IgG and anti-rabbit IgG were ordered from Beyotime (Shanghai, China).

### 2.3. Cytotoxicity Assay

The cytotoxicity of T-4-O was evaluated using a CCK-8 assay. Briefly, RAW264.7 cells were seeded at 5 × 10^4^ per well in 96-well plates and grown at 37 °C for 12 h. The next day, the medium was changed to DMEM/2% FBS supplemented with T-4-O (0, 12.5, 25, 50, 100, 200, 400, and 800 μmol/L), and the cells were cultured for 24 h. CCK-8 reagent was then added to each well, and the cells were incubated for 1 h at 37 °C. The absorbance was measured at 450 nm with a microplate reader and expressed as a percentage of the control value. The mean optical density (OD) values from six wells per treatment were used as the cell viability index.

### 2.4. RT-qPCR

Real-time quantitative PCR (RT-qPCR) was performed using AceQ Universal SYBR qPCR Master Mix on a LightCycler 480 system. The reactions were performed in a final volume of 10 μL, using 1 μL of diluted cDNA and 0.1 μL each of the forward and reverse primers. Amplification was performed using the following cycling conditions: 95 °C for 10 s, 60 °C for 30 s, and 72 °C for 30 s, with a total of 40 cycles of amplification. The expression of mRNA was normalized by using *GAPDH* mRNA as a control and calculated based on the 2^−ΔΔCt^ method. The primer sequences are listed in Table 1.

### 2.5. Oxygen Consumption Rate and Extracellular Acidification Rate Measurements

To measure the oxygen consumption rate (OCR) and extracellular acidification rate (ECAR), 5 × 10^4^ cells were seeded the day before the experiment on an XFe24 cell culture microplate. The next day, cells were washed twice in phosphate-buffered saline (PBS). Cells were incubated with T-4-O for 2 h, then stimulated with LPS for 6 h. To eliminate residues of carbonic acid from the medium, cells were washed with XF Base Medium (Agilent Technology, Santa Clara, CA, USA) and incubated for at least 30 min at 37 °C with atmospheric CO_2_ in a non-humidified incubator. The OCR and ECAR were assessed with an XF96 extracellular flux analyzer (Seahorse; Agilent). Glucose (10 mM), oligomycin (1 μM), and 2-deoxy-d-glucose (2-DG) (Sigma, 100 mM) were injected onto the plate sequentially. Oligomycin (1 μM), carbonyl cyanide-p-trifluoromethoxyphenylhydrazone (FCCP, 2.5 μM), rotenone (1 μM), and antimycin A (1 μM) were also injected onto the plate sequentially. Data were analyzed using XF Wave (v.2.6) software.

### 2.6. Metabolomics Analysis

RAW264.7 cells were obtained in the logarithmic growth phase and inoculated in culture dishes with a diameter of 10 cm at a cell density of 5 × 10^6^ cells/well. These cells were incubated for 24 h and then were divided into four groups and pretreated with different drugs for 2 h: control group (untreated cells), experimental group (100 μmol/L T-4-O), model group (1 μg/mL LPS), and treatment group (100 μmol/L T-4-O and 1 μg/mL LPS). The cells were stimulated with LPS for 6 h. The cells were collected according to the requirements, rapidly frozen in liquid nitrogen for 15 min, and immediately stored at −80 °C before transportation to BGI Genomics (Shenzhen, China).

The original LC-MS/MS data obtained from BGI Genomics were imported into Compound Discoverer (v.2.0) software (Thermo Fisher, Waltham, MA, USA) to acquire matched and aligned peak data. The obtained peak areas were normalized and then imported into SIMCA-P (v.13.0) software for principal component analysis (PCA), partial least squares discriminant analysis (PLS-DA), and orthogonal partial least squares discriminant analysis (OPLS-DA). Subsequently, the OPLS-DA model VIP > 1 and the *t* test (*p* < 0.05) were used to filter differential metabolites (DAMs) between the two groups. The Kyoto Encyclopedia of Genes and Genomes (KEGG) pathway enrichment analysis of the identified DAMs was carried out using MetaboAnalyst (v.5.0) software.

### 2.7. Western Blot

RAW264.7 cells were seeded in six-well plates, and the density of each well was adjusted to 9 × 10^5^. Then, the cells were placed in four groups after 24 h of culture: control group, experimental group (final concentration of 100 μmol/L terpinene-4-ol), model group (final concentration of 1 μg/mL LPS), and treatment group (final concentration of 100 μmol/L terpinene-4-ol and 1 μg/mL lipopolysaccharide). After 2 h of drug pretreatment, DMEM with or without LPS was used to incubate the cells for 6 h. Proteins from the cells were lysed by using ice-cold radio immunoprecipitation assay (RIPA) buffer and centrifuged at 14,000× *g* for 5 min. The protein concentrations of the samples were determined using a bicinchoninic acid assay kit according to the manufacturer’s instructions. A quantity of 15 µg of lysates was subjected to 10% sodium dodecyl sulfate–polyacrylamide gel electrophoresis and transferred onto polyvinylidene difluoride (PVDF) membranes. The PVDF membranes were blocked with 5% fat-free milk for 2 h and incubated with the corresponding primary anti-bodies at 4 °C overnight (mTOR, p-mTOR, AMPK, p-AMPK, NF-κB-p65, p-NF-κB-p65, IκBα, p-IκBα, α-tubulin, and GAPDH). After washing, the membranes were incubated with the appropriate HRP-conjugated secondary antibody for 2 h at room temperature. Protein bands were subsequently detected with an enhanced chemiluminescence kit, and the band intensities were analyzed using ImageJ (v.1.8.0) software.

### 2.8. Statistical Analyses

All statistical analyses were conducted using GraphPad Prism (v.7.0). The results are expressed as mean ± standard error of the mean of at least three independent experiments. Differences between groups were evaluated using one-way or two-way analysis of variance (ANOVA).

## 3. Results

### 3.1. T-4-O Inhibits mRNA Expression of Inflammatory Factors in Mouse RAW264.7 Macrophages

**Figure 1:**
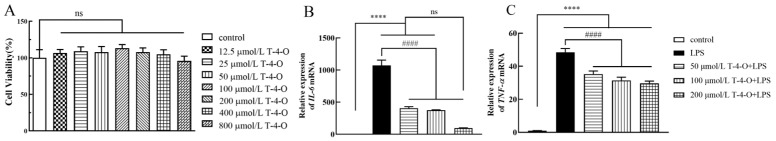
Effect of terpine-4-ol on mRNA expression of inflammatory cytokines. (**A**) Effect of terpinene-4-ol on the viability of mouse RAW264.7 macrophages. (**B**) Effect of terpinene-4-ol on *IL-6* mRNA expression levels in mouse RAW264.7 macrophages induced by LPS. (**C**) Effect of terpinene-4-ol on *TNF-α* mRNA expression levels in mouse RAW264.7 macrophages induced by LPS. ns, not significantly different from the control group at *p* > 0.05; ****, significantly different from the control group at *p* < 0.0001; ####, significantly different from the LPS group at *p* < 0.0001.

In order to determine whether T-4-O of different concentrations had toxic effects on mouse RAW264.7 macrophages, seven concentrations were used, 12.5 μmol/L, 25 μmol/L, 50 μmol/L, 100 μmol/L, 200 μmol/L, 400 μmol/L, and 800 μmol/L, with a blank control group without T-4-O. After 24 h of treatment, a CCK-8 assay showed that relative to that of the blank control group, cell viability was 106.60%, 109.24%, 107.85%, 113.19%, 107.86%, 104.89%, and 95.94%, respectively (Figure 1A), and there were no significant differences in cell viability in the groups treated with various concentrations of T-4-O (*p* > 0.05). These results showed that a concentration of less than 800 μmol/L T-4-O did not affect the viability of RAW264.7 cells. Next, we analyzed the effects of 50 μmol/L, 100 μmol/L, and 200 μmol/L T-4-O on the LPS-induced inflammatory cytokines *IL-6* (Figure 1B) and *TNF-α* (Figure 1C). The mRNA expression of *IL-6* and *TNF-α* in the LPS model group increased significantly, compared with the blank control group (*p* < 0.001). The T-4-O treatment and LPS model groups showed that *IL-6* and *TNF-α* mRNA expression decreased in a dose-dependent manner. These results demonstrated that T-4-O can improve inflammatory responses in cells by inhibiting the expression of LPS-induced inflammatory cytokines *IL-6* and *TNF-α*.

### 3.2. Changes in Energy Metabolism in LPS-Induced Macrophages by T-4-O

**Figure 2:**
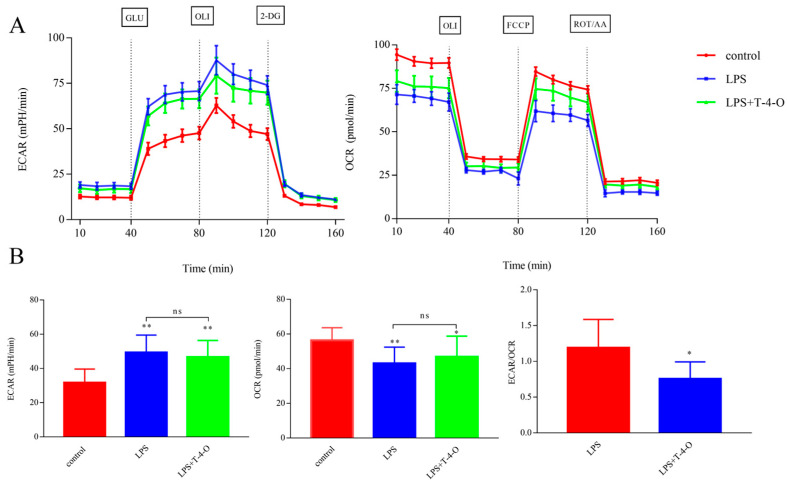
Terpinene-4-ol regulates LPS-induced glycolysis in macrophages. (**A**) The ECAR and OCR in RAW264.7 cells stimulated with LPS or LPS + T-4-O for 6 h was measured with a glycolysis stress test kit. (**B**) Quantification of the ECAR, OCR, and ECAR/OCR ratio in RAW264.7 cells. **, *p* < 0.01; *, *p* < 0.05; ns, not significant.

Energy metabolism is intimately associated with macrophage-mediated inflammatory responses. In order to evaluate energy metabolism changes after macrophages were stimulated by LPS, we measured the ECAR and OCR. The results showed that the ECAR increased and OCR decreased after mouse RAW264.7 cells were stimulated by LPS (Figure 2A,B). T-4-O could improve the inflammatory response induced by LPS. Compared with LPS treatment, treatment with T-4-O decreased the ECAR by 5.53%, increased the OCR by 8.66%, and decreased the ECAR/OCR ratio significantly (Figure 2B). These results showed that glucose metabolism in RAW264.7 cells changed from glycolysis to oxidative phosphorylation.

### 3.3. Metabolomics PCA and OPLS-DA

**Figure 3:**
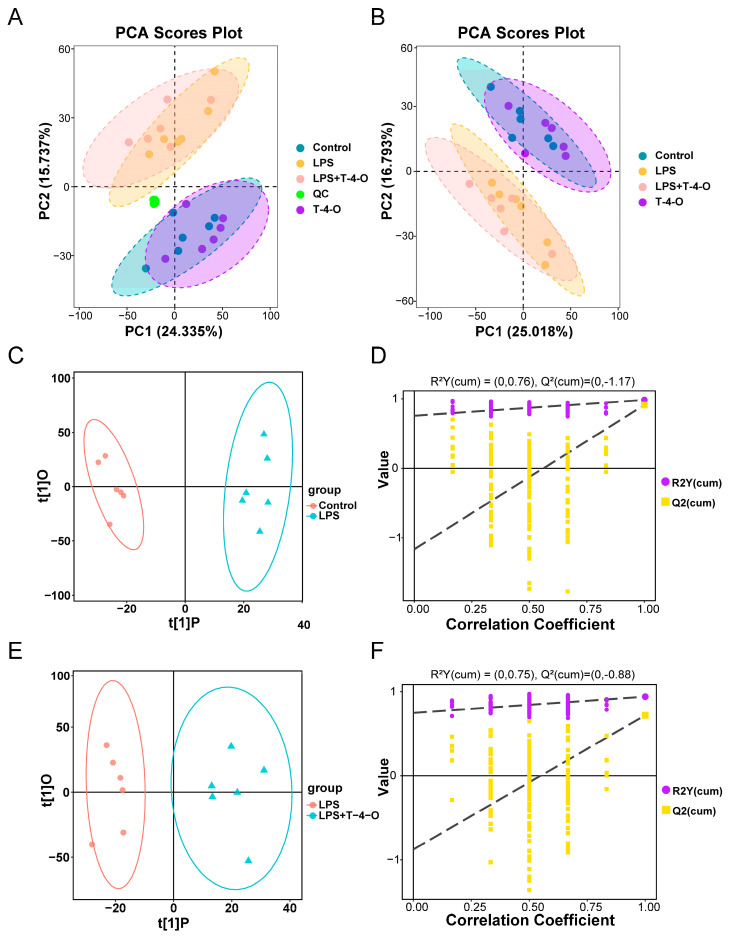
The analysis results of metabonomic data. (**A**) PCA score plots of cell samples from QC, Control, T-4-O, LPS, and LPS + T-4-O groups. (**B**) PCA score plots of cell samples from Control, T-4-O, LPS, and LPS + T-4-O groups. (**C**) OPLS-DA scatter plots in Control and LPS groups. (**D**) Model validation results in Control and LPS groups. (**E**) OPLS-DA scatter plots in LPS and LPS + T-4-O groups. (**F**) The model validation results in LPS and LPS + T-4-O groups.

PCA was used to compare the metabolome data of all samples. As shown in Figure 3A,B, the QC samples clustered tightly, indicating that the systematic error was low, the experiment was repeatable, and the data were eligible. Between experimental samples, the control and T-4-O groups were clustered close to each other, while the LPS and LPS + T-4-O groups were clustered together, showing that there were metabolite differences between normal mice and LPS-induced mice. On the basis of determining inter-group metabolic differences, we further employed OPLS-DA to screen for inter-group DAMs, including the control group vs. the LPS group and the LPS group vs. the LPS + T-4-O group. The OPLS-DA models of the various groups had an R^2^Y ≥ 0.75 (Figure 3C–F), indicating that the constructed statistical model was explanatory and had predictive power, and can be used for further analysis.

### 3.4. Differentially Expressed Metabolite Screening and Enriched Pathway Analysis

**Figure 4:**
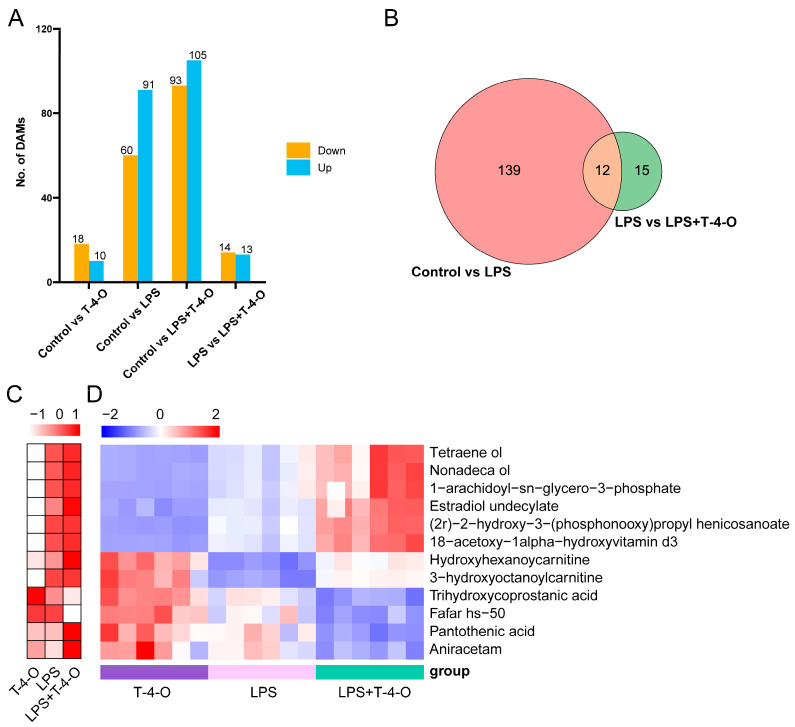
Identification and analysis of DAMs. (**A**) DAMs number for different samples. (**B**) Venn diagram of DAMs regarding control vs. LPS and LPS vs. LPS + T-4-O. (**C**) Twelve potential biomarkers for T-4-O treatment, LPS treatment, and LPS + T-4-O treatment. Variable importance in projection (VIP). (**D**) Expression heatmaps of 12 DAMs. The expression value of each DAM was normalized by Z-score. Tetraene ol represents (1s,3r,5z,7e,22e)-9,10-secoergosta-5,7,10,22-tetraene-1,3,25,28-tetrol; Nonadeca ol represents (1s,2r,5r,6r,10r,13s,15s)-5-[(2r,3e,5r)-5,6-dimethyl-3-hepten-2-yl]-6,10-dimethyl-16,17-dioxapentacyclo[13.2.2.01,9.02,6.010,15]nonadeca-8,18-dien-13-ol.

The OPLS-DA model was used to identify DAMs with variable importance in projection (VIP) ≥ 1, *p* < 0.05, and fold change (FC) > 1.2 or FC < 0.83. As shown in Figure 4A, 151 DAMs were obtained by comparing the control and LPS groups, of which, 91 DAMs were up-regulated and 60 DAMs were down-regulated. These DAMs included spermine, L-proline, L-glutathione (reduced), pantothenic acid, and taurine. There were 13 DAMs that were up-regulated and 14 DAMs that were down-regulated between the LPS and LPS + T-4-O groups, including L-glutamine, L-threonine, pyridoxine, Dl-homoserine, and condore. Venn diagrams were used to compare the common DAMs between the two groups of samples. A total of 12 DAMs were identified across the two comparisons (control vs. LPS groups, and LPS vs. LPS + T-4-O groups) (Figure 4B), which included pantothenic acid, hydroxyhexanoycarnitine, and 1-arachidoyl-sn-glycero-3-phosphate. Among them, four DAMs were down-regulated after T-4-O treatment (Fafar hs-50, trihydroxycoprostanic acid, pantothenic acid, and aniracetam), while eight DAMs were up-regulated (Tetraene ol, Nonadeca ol, 1-arachidoyl-sn-glycero-3-phosphate, 18-acetoxy-1-alpha-hydroxyvitamin d3, estradiol undecylate, (2r)-2-hydroxy-3-(phosphonooxy) propyl henicosanoate, hydroxyhexanoycarnitine, and 3-hydroxyoctanoylcarnitine). We postulate that these 12 metabolites may be associated with the T-4-O improvement in mouse inflammation. Overall, compared to the control group, the number of DAMs significantly increased after the addition of LPS, leading to tremendous changes in metabolites and metabolic pathways in the mouse cells. We also prepared differential expression heat maps and VIP result graphs for the 12 DAMs (Figure 4C,D).

**Figure 5:**
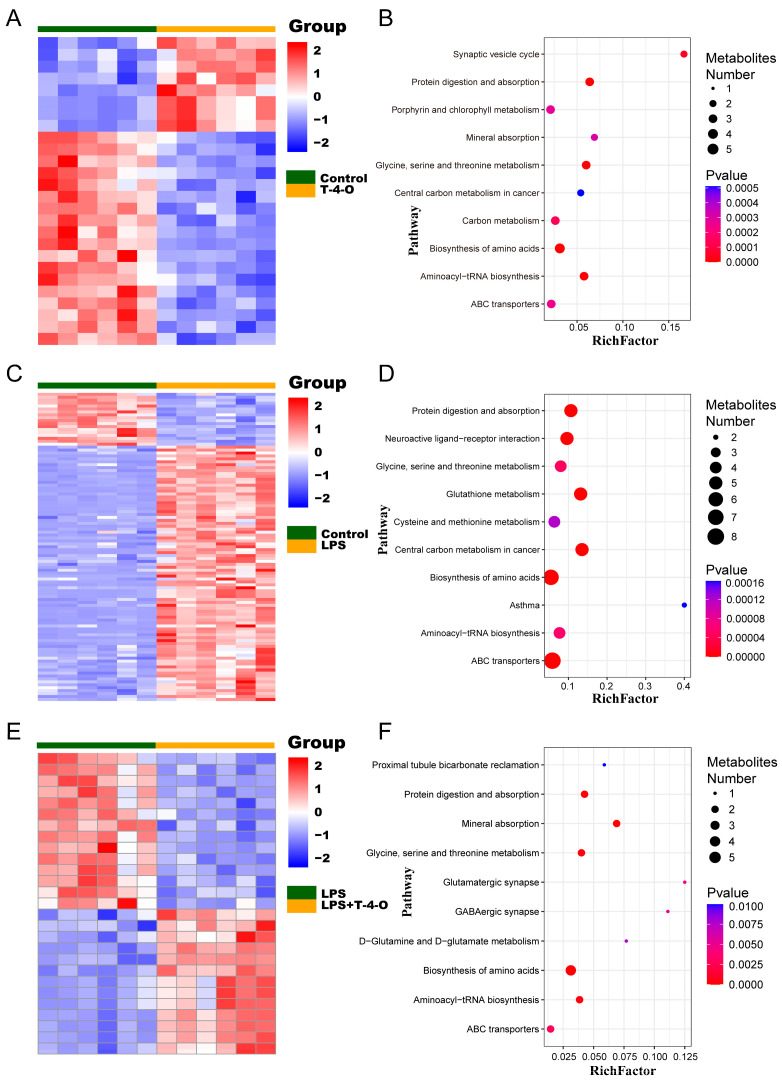
Expression heatmaps and KEGG functional enrichment analysis results of DAMs between different treatments. (**A**) Expression heatmaps of DMAs between control and T-4-O. (**B**) KEGG functional enrichment analysis of DAMs between control and T-4-O. (**C**) Expression heatmaps of DMAs between control and LPS. (**D**) KEGG functional enrichment analysis of DAMs between control and LPS. (**E**) Expression heatmaps of DMAs between LPS and LPS + T-4-O. (**F**) KEGG functional enrichment analysis of DAMs between LPS and LPS + T-4-O.

Subsequently, the DAMs were entered into the MetaboAnalyst website for KEGG pathway enrichment analysis (Figure 5A–F). Compared with the control group, LPS-group DAMs were mainly enriched in glutathione metabolism, cysteine metabolism, and methionine metabolism (Figure 5C,D). In the T-4-O group, DAMs were mainly enriched in metabolic pathways of glutamine and glutamate metabolism and glycine, serine, and threonine metabolism (Figure 5E,F). The results showed that the anti-inflammatory effects of T-4-O may be achieved through regulating the metabolism of different amino acids to maintain glutamine and glutamate metabolism to decrease the release of inflammatory factors.

### 3.5. Analysis of the Effects of T-4-O on Glutamine Metabolism-Regulated Genes

**Figure 6:**
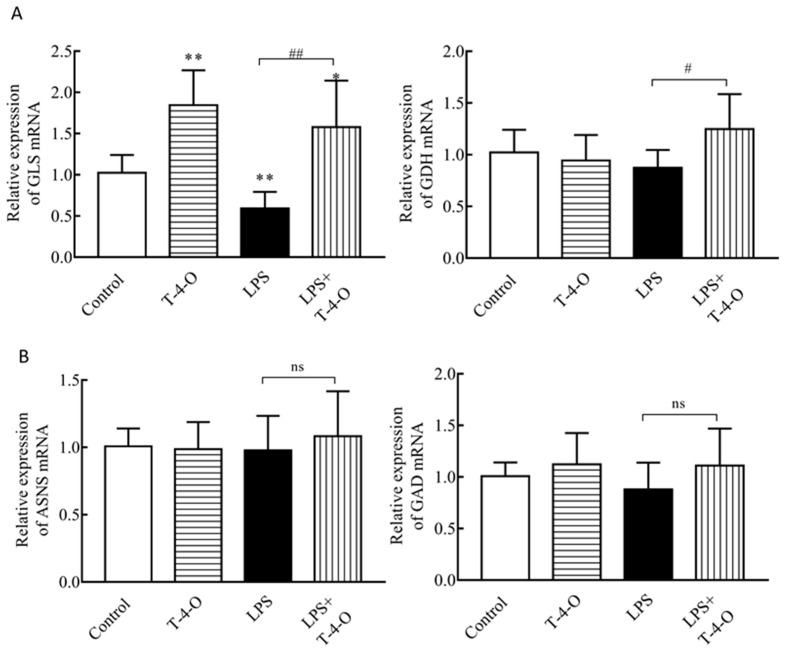
T-4-O enhances glutamine metabolism. (**A**) Changes in mRNA expression levels of *GLS* and *GDH*. (**B**) Changes in mRNA expression levels of *ASNS* and *GAD*. ns, no significant difference; *, *p* < 0.05, **, *p* < 0.001 significantly different from the control group; #, *p* < 0.05, ##, *p*< 0.001 significantly different from the LPS group.

The above results showed that T-4-O increased the glutamine catabolism inhibited by LPS. There are two glutamine metabolic processes: one is dependent on glutaminase and glutamate dehydrogenase to produce α-ketoglutaric acid, and the other is dependent on asparagine synthetase and a γ-aminobutyric acid metabolic shunt to produce ATP. Therefore, we measured mRNA changes in glutamine metabolism enzymes. As shown in Figure 6A,B, *GLS* and *GDH* mRNA significantly increased (*p* < 0.01) in cells treated with LPS + T-4-O, but there was no significant change in *ASNS* or *GAD* mRNA. This result showed that T-4-O promoted glutamine metabolism to convert glutamic acid into α-ketoglutaric acid instead of converting glutamic acid into γ-aminobutyric acid, thereby increasing the oxidative phosphorylation level in cells.

### 3.6. Effects of T-4-O on LPS-Regulated mTOR, AMPK, and NF-κB Signaling Pathway-Related Proteins

**Figure 7:**
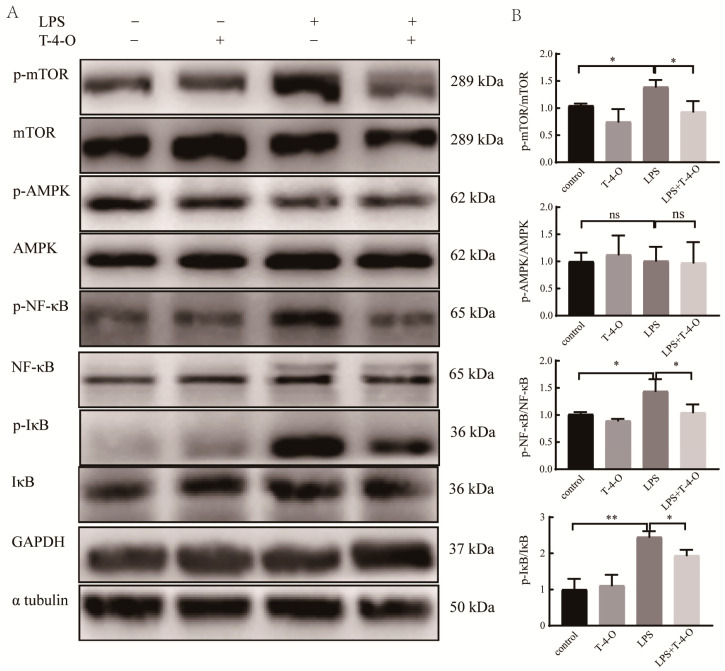
The expression of related signaling pathway proteins induced by LPS. (**A**) Western blotting detection of protein in RAW264.7 cells. (**B**) Gray values of Western blotting bands were analyzed by ImageJ. ns, no significant difference; *, *p* < 0.05, **, *p* < 0.01.

Glutamine metabolism has many functions in cells, such as energy metabolism and autophagy. Some studies found that glutamine metabolism was regulated by mTOR, which could sense the microenvironment energy demand and regulate cell metabolic pathways, thereby modifying NF-κB activity and regulating inflammation [13]. AMPK is a cellular energy sensor that helps coordinate glucose and lipid metabolism. Therefore, the effects of T-4-O on LPS-induced mTOR, AMPK, and NF-κB signaling pathway-related proteins were examined. As shown in Figure 7A,B, the mTOR protein phosphorylation (p-mTOR) level in the model group was significantly increased compared with the blank control group, and the IκB and NF-κB protein phosphorylation levels (p-IκB and p-NF-κB) were significantly increased as well (*p* < 0.05), whereas there was no significant difference in the AMPK protein phosphorylation (p-AMPK) level. This result showed that LPS could activate the mTOR and NF-κB signaling pathways. Compared with the model group, p-mTOR, p-IκB, and p-NF-κB were significantly decreased in the LPS + T-4-O group (*p* < 0.05), indicating that T-4-O may inhibit the mTOR and NF-κB activity to improve LPS-induced inflammatory responses.

## 4. Discussion

Many studies have found that TTO can inhibit LPS- [14,15], bacteria- [16], and Indiana vesiculovirus- [17] induced host cell inflammation. TTO has diverse components, which mainly include T-4-O, γ-terpinene, α-terpineol, and 1,8-cineole [18]. Among them, T-4-O is an active ingredient of TTO and is one of the most bioactive substances. Studies have shown that T-4-O inhibited bacteria and parasitic worm-induced inflammation [19,20,21]. In this study, the mRNA transcript levels of *IL-6* and *TNF-α* significantly increased in cells of the LPS model but decreased significantly when T-4-O was added. The results further demonstrated the anti-inflammatory effects of T-4-O.

Studies have shown that macrophages obtain energy through glycolysis during inflammatory activation and that the inhibition of glycolysis can inhibit inflammation [22,23,24]. Our study found that the ECAR increased, OCR decreased, and glycolysis increased during glucose metabolism when RAW264.7 cells were stimulated by LPS. After T-4-O treatment, the ECAR/OCR ratio significantly decreased, indicating that glycolysis decreased and oxidative phosphorylation increased. These results showed that T-4-O regulated energy metabolism changes to promote the conversion to oxidative phosphorylation, which ameliorates the LPS-induced inflammation in macrophages.

Non-targeted metabolomics and pharmacological research were combined to analyze the metabolism characteristics of the individual drug interventions, identify DAMs, and predict the possible metabolic processes and mechanisms in which these DAMs participated. This provided theoretical support for further pharmacological analysis of the drugs. Non-targeted metabolomics was employed. Compared with the control, 151 metabolites were identified after LPS treatment. Further enrichment analysis found that DAMs were mainly enriched in glutathione metabolism, cysteine metabolism, and methionine metabolism. After T-4-O treatment, DAMs were mainly enriched in the metabolic pathways of glutamine and glutamate metabolism and glycine, serine, and threonine metabolism. After glutamine enters the cytoplasm, it is first converted to glutamic acid by *GLS* or converted to asparagine and glutamic acid through *ASNS* catalysis of aspartic acid and glutamine. Then, glutamic acid is converted to α-ketoglutaric acid (α-KG) by *GDH*. α-KG participates in the tricarboxylic acid cycle (TCA cycle) and supports the oxidative phosphorylation (OXPHOS) pathway after entering the mitochondria [25,26]. Glutamic acid can also be converted to inhibitory neurotransmitter γ-aminobutyric acid (GABA) and ATP by GAD, which regulates the mTOR signaling pathway [27]. Metabolomics results showed that α-KG levels extremely significantly increased, but there was no change in the GABA level after T-4-O treatment. qPCR was used to test the mRNA changes in related genes. The results showed that *GLS* and *GDH* mRNA expression extremely significantly increased after T-4-O treatment, but there were no significant changes in *ASNS* or *GAD* mRNA expression. These results were consistent with the metabolomics results and showed that T-4-O treatment promoted glutamine metabolism to convert glutamic acid to α-ketoglutaric acid instead of γ-aminobutyric acid, thereby increasing the oxidative phosphorylation level in cells.

**Figure 8:**
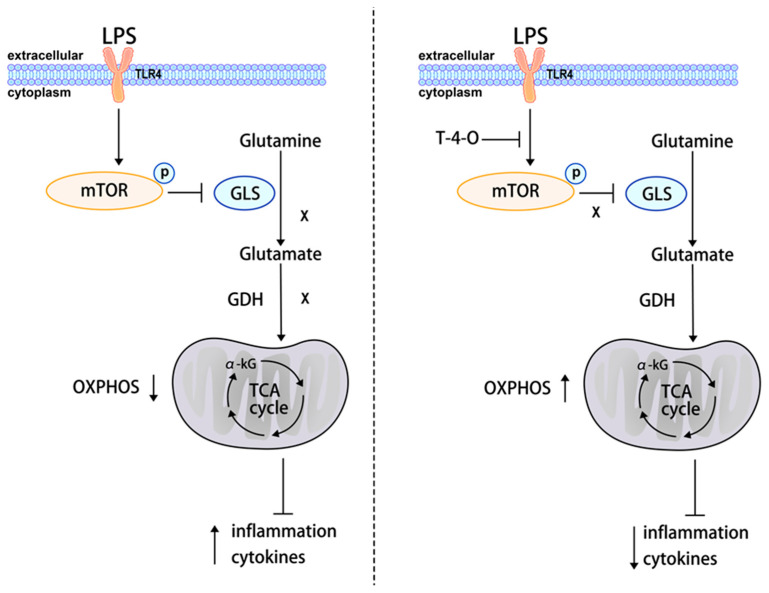
A model of treating LPS-induced inflammation with T-4-O.

mTOR regulates inflammation, reactive oxygen species, DNA damage, and protein homeostasis and inhibits autophagy. mTOR also guides the conversion from aerobic metabolism (oxidative phosphorylation) to anaerobic metabolism (glycolysis) and promotes the expression of many inflammatory factors (such as *IL-6* and *TNF-α*) through NF-κB [28]. NF-κB is a protein complex that controls DNA transcription and is nearly ubiquitous in all animal cells. NF-κB participates in cell stress and cytokine and immune responses and is an important regulator of immune responses to inflammation and infection [29,30,31]. IκB binds to NF-κB dimers in the cytoplasm, masking the nuclear localization signal of NF-κB to prevent it from entering the nucleus and binding to DNA. This causes NF-κB to exist in an inactive state in the cytoplasm. Our study found that LPS promoted mTOR activation and IκB phosphorylation and degradation, which increased NF-κB phosphorylation but with no significant effect on the AMPK level. After T-4-O treatment, the mTOR and NF-κB phosphorylation levels significantly decreased. This showed that T-4-O regulated mTOR phosphorylation to inhibit the activation of NF-κB, thus ameliorating inflammatory responses. mTOR inhibition was found to improve the expression level of intracellular *GLS*, thereby increasing glutamine metabolism [32,33,34]. mTOR was also found to positively regulate *GLS* transcription [35,36], indicating that there are many pathways by which mTOR regulates *GLS*. Our results showed that *GLS* expression and glutamine metabolism increased after mTOR was inhibited by T-4-O. Therefore, we propose that the mechanism by which T-4-O improves LPS-induced macrophage inflammation is as follows: T-4-O inhibits mTOR phosphorylation to increase *GLS* expression, thereby promoting glutamine metabolism. The α-KG synthesis is increased and enters the TCA cycle to increase oxidative phosphorylation levels and inhibit activation of the NF-κB signaling pathway. This mechanism improves the LPS-induced macrophage inflammatory responses (Figure 8).

## 5. Conclusions

The results of this study showed that T-4-O significantly inhibited LPS-induced inflammatory cytokine expression in RAW264.7 macrophages. T-4-O inhibited mTOR phosphorylation to regulate glutamine metabolism and increase cell oxidative phosphorylation levels, thereby improving LPS-induced cell inflammatory responses. In this study, the mechanism of T-4-O in improving LPS-induced macrophage inflammation was analyzed from a metabolic and experimental perspective, providing a theoretical basis for subsequent inflammation research. Additionally, the anti-inflammatory effects of T-4-O could also facilitate its subsequent application in the field of food additives.

## Figures and Tables

**Table 1:** Primers for qPCR.

Gene	Forward Primer (5′–3′)	Reverse Primer (5′–3′)
*GAPDH*	CGTGCCGCCTGGAGAAACCTG	AGAGTGGGAGTTGCTGTTGAAGTCG
*TNF-α*	GTGGAACTGGCAGAAGAGGC	AGACAGAAGAGCGTGGTGGC
*IL-6*	ACAGAAGGAGTGGCTAAGGAC	GCTTAGGCATAACGCACTAGG
*GLS*	CAGTCTGAACGAGAAAGTGGAGA	ATCCCAACCATGTCTGTGCC
*GDH*	TCAGTTGGAATCAGCCCCTT	GTGACTGACTGCTCCTGACT
*ASNS*	GAAACTCTTCCCAGGCTTTGAC	TTCAGCAGAGAGGCAGCAAC
*GAD*	GGCTCCGGCTTTTGGTCCTTC	TGCCAATTCCCAATTATACTCTTGA

## Data Availability

The original contributions presented in the study are included in the article, further inquiries can be directed to the corresponding authors.
